# Molecular Mechanisms of Bacterial Resistance to Metal and Metal Oxide Nanoparticles

**DOI:** 10.3390/ijms20112808

**Published:** 2019-06-08

**Authors:** Nereyda Niño-Martínez, Marco Felipe Salas Orozco, Gabriel-Alejandro Martínez-Castañón, Fernando Torres Méndez, Facundo Ruiz

**Affiliations:** 1 Facultad de Ciencias, Universidad Autónoma de San Luis Potosí, San Luis Potosí Cp 78210, Mexico; nereyda.nino@uaslp.mx (N.N.-M.); ruizfacundo1@gmail.com (F.R.); 2 Facultad de Estomatología, Universidad Autónoma de San Luis Potosí, San Luis Potosí Cp 78210, Mexico; mtzcastanon@fciencias.uaslp.mx (G.-A.M.-C.); fernando.torres@uaslp.mx (F.T.M.)

**Keywords:** bacteria, resistance, nanoparticles

## Abstract

The increase in bacterial resistance to one or several antibiotics has become a global health problem. Recently, nanomaterials have become a tool against multidrug-resistant bacteria. The metal and metal oxide nanoparticles are one of the most studied nanomaterials against multidrug-resistant bacteria. Several in vitro studies report that metal nanoparticles have antimicrobial properties against a broad spectrum of bacterial species. However, until recently, the bacterial resistance mechanisms to the bactericidal action of the nanoparticles had not been investigated. Some of the recently reported resistance mechanisms include electrostatic repulsion, ion efflux pumps, expression of extracellular matrices, and the adaptation of biofilms and mutations. The objective of this review is to summarize the recent findings regarding the mechanisms used by bacteria to counteract the antimicrobial effects of nanoparticles.

## 1. Introduction

The increase in bacterial resistance to one or several antibiotics has become a global health problem [1]. The antibiotics were discovered in the 1920s. Since then, the discovery and use of new antibiotics have been accompanied by the appearance of bacterial resistance to them. The great genetic flexibility of the bacteria and the selection pressure exerted by the use of the antibiotics are responsible for the appearance and perpetuation of antibiotic resistance. Likewise, the variety of mechanisms of resistance to the different types of antibiotics has increased over time [2].

Recommendations have been suggested to avoid the constant appearance of resistance to antibiotics; these recommendations include: Improve sanitation and prevent the spread of infection, reduce unnecessary use of antimicrobials, improve global surveillance of drug resistance and antimicrobial consumption and promote rapid diagnostics techniques to reduce unnecessary use of antimicrobials. Likewise, new options for compounds with antimicrobial properties are continually being sought [3].

Recently, nanomaterials have become a tool against multidrug-resistant bacteria. These nanomaterials can be used as nano-drugs that can act individually or in synergism with antimicrobial compounds against resistant bacteria. Nanomaterials are also used as drug delivery systems that provide greater therapeutic efficacy and enhanced physicochemical characteristics. The metal and metal oxide nanoparticles are one of the most studied nanomaterials against multidrug-resistant bacteria. These nanoparticles can be synthesized from metals and metal oxides as gold, silver, titanium, copper, zinc, and aluminum; as well as silver oxide, copper oxide, magnesium oxide, calcium oxide, and zinc oxide.

Several in vitro studies report that metal nanoparticles have antimicrobial properties against a broad spectrum of bacterial species. This is due to the special characteristics of metal and metal oxide nanoparticles. These special characteristics include increased surface/volume ratio of the nanoparticles with a decrease in the particle size, nanoparticle stability, van der walls forces or hydrophobic interactions and, electrostatic attraction. These characteristics allow nanoparticles to exercise their antimicrobial activity through multiple mechanisms. These antimicrobial mechanisms include damage to the membrane and bacterial cell wall, damage to proteins and internal components of bacteria, release of ions, DNA damage and oxidative stress [4].

The great diversity of antimicrobial mechanisms exerted by nanoparticles suggests that bacteria are unlikely to generate resistance to nanoparticles [5]. However, until recently, the bacterial resistance mechanisms to the bactericidal action of the nanoparticles had not been investigated. Some of the recently reported resistance mechanisms include electrostatic repulsion, ion efflux pumps [6], expression of extracellular matrices [7], mutations [8] and, biofilm adaptation.

The objective of this review is to summarize the recent findings regarding the mechanisms used by bacteria to counteract the antimicrobial effects of nanoparticles.

## 2. Defense Mechanisms against Different Sizes of Metal and Metal Oxide Nanoparticles.

As mentioned above, the smaller the size of the nanoparticles, the greater their surface/volume ratio. The increase of the surface/volume ratio of the nanoparticles improves their ability to interact with different components of bacteria and exert their antimicrobial mechanisms [9]. For example, Agnihotri et al. synthesized silver nanoparticles (Ag NPs) with approximate sizes of 5, 7, 10, 15, 20, 30, 50, 63, 85, and 100 nm. They observed that the antibacterial effect of the different nanoparticle samples depended on dose and size. The nanoparticles with sizes of 5, 7 and 10 nm showed the highest bactericidal activity. [10]. In another study, Azam et al. synthesized three (3) different types of metal oxide nanoparticles, with three different sizes each. The zinc oxide nanoparticles (ZnO NPs) had a size of 18 nm, the copper oxide nanoparticles (CuO NPs) had a size of 22 nm and, the iron oxide nanoparticles (Fe_2_O_3_ NPs) had a size of 28 nm. The bactericidal power of these three types of nanoparticles was evaluated against Gram-positive (*Bacillus subtilis* and *Staphylococcus aureus*) and Gram-negative (*Pseudomonas aeruginosa* and *Escherichia coli*) bacteria. Of the three types of nanoparticles, those with the smallest size (ZnO NPs—18 nm) had the highest bactericidal activity, while the nanoparticles with the largest size (Fe_2_O_3_ NPs–28 nm) showed the lowest bactericidal activity. Some studies suggest that the size of nanoparticles is one of the main factors of their antimicrobial power, although this may also depend on the type of synthesis, precursors and parameters used. For example, Raza et al. synthesized silver nanoparticles with different size and shape. They synthesized Ag NPs with spherical shape and diameters from 15 to 90 nm, while the triangular-shaped Ag NPs had approximate sizes at 150 nm. The evaluation of the antibacterial effect of both types of nanoparticles showed that the spherical and smaller nanoparticles had the greatest antibacterial power against Gram-negative bacteria [11]. Helmlinger et al. synthesized Ag NPs with four different shapes and different sizes each. Spherical nanoparticles with sizes of 40–80 nm and 120–140 nm, rods with 80–120 nm, platelets with 20–60 nm and cubes with 140–180 nm. The bactericidal effect of these nanoparticles against *Staphylococcus aureus* seems not to be determined by their shape but by the ability of the nanoparticles to release small-sized elements, such as Ag^+^ ions, that can penetrate the defenses of bacteria. This is due to the fact that the platelets showed the highest bactericidal effect due to a greater release of silver ions. In addition, the platelets showed the highest surface/volume ratio of all samples. Therefore, size remains one of the important factors of the bactericidal effect of different types of nanoparticles [12]. In general, several studies have shown that nanoparticles smaller than 10 nm can penetrate to the interior of bacterial cells, and this increases their bactericidal activity [13]. While in general, nanoparticles greater than 10 nm cannot penetrate the interior of bacteria, which decreases its bactericidal power [14,15].

**Figure 1:**
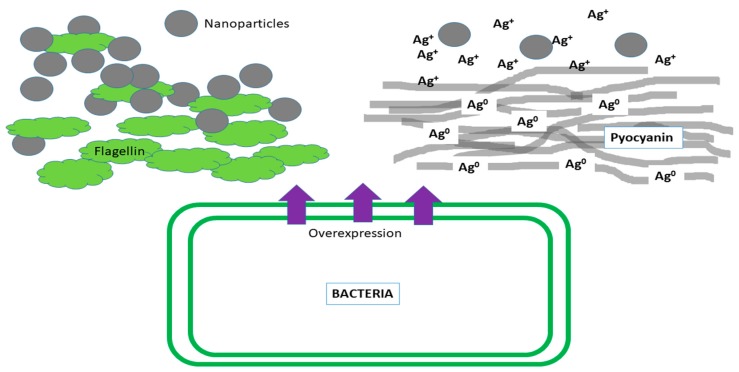
The defense mechanisms of the bacteria against nanoparticles include the production of extra-cellular substances (like flagellin) with the ability to agglomerate nanoparticles, and the production of pigments (like pyocyanin) capable of inactivating the ions released by the nanoparticles.

The literature suggests that bacteria have two mechanisms that allow them to defend themselves from small and large nanoparticles. The mechanism responsible for preventing the antimicrobial effect of nanoparticles greater than 10 nm—that tend to react with the wall and bacterial membrane causing its disintegration and the death of the bacterial cell—is the production of extracellular matrix responsible for agglomerating and deactivating of the nanoparticles (Figure 1). However, a recent study shows that some bacteria (*Escherichia coli* 013 and *Pseudomonas aeruginosa* CCM 3955) are capable of overexpressing a flagellin matrix that causes the agglomeration of 20 nm silver nanoparticles and avoids their direct contact with the bacteria [16]. Faghihzadeh et al. also reported the adaptation of *E. coli* to Ag NPs through the production of extracellular substances (ECS). These ECS were able to modify the size and zeta potential of the nanoparticles by causing their agglomeration. In addition, the composition of the ECS depended on the growth conditions used [17]. On the other hand, the interaction of the nanoparticles in pathophysiological environments leads to the formation of biomolecule coronas (similar to the ECS) around the nanoparticles that prevent their contact with the pathogens and decrease their bactericidal activity [18].

The mechanism responsible for preventing the antimicrobial effect of nanoparticles smaller than 10 nm, and the silver ions released by larger nanoparticles; is the change of expression of genes and mutations that cause a decrease in the possible entry pathways of the nanoparticles to the bacteria. For example, nanoparticles smaller than 10 nm can enter the bacteria and react with a variety of internal components. However, bacteria exposed to non-lethal concentrations of Ag NPs showed an increase in their resistance due to mutations that caused a downregulation of several genes [8]. This change of expression of genes resulted in the downregulations of porins, preventing the entry of nanoparticles into the bacteria [19]. Hachicho et al. reported that *Pseudomonas putida* was able to change the conformation of the unsaturated fatty acids present in its membrane. This change of conformation produces a change in the fluidity of the membrane which makes it less permeable and prevents the passage of nanoparticles and ions [20].

## 3. Defense Mechanisms against the Surface Charge of Metal and Metal Oxide Nanoparticles

Although some articles seem to show that the size of metal and metal oxide are the most important property in terms of its antimicrobial effect [10]. Other articles give more importance to the electric charge present on the surface of the different types of metal and metal oxide nanoparticles [21]. For example, Stoimenov et al. synthesized three different types of magnesium oxide nanoparticles (MgO NPs) and evaluated their bactericidal action against endospores, Gram-positive and Gram-negative bacteria. They determined that the nanoparticles had bactericidal action against the three types of microorganisms tested and, that this effect was due to the electrostatic attraction of the nanoparticles with a positive charge and the negatively charged pathogens [22]. Haggstrom et al. tested the antimicrobial activity of a large variety of three types of metal oxide nanoparticles (Al_2_O_3_ NPs, TiO_2_ NPs, and CeO_2_ NPs). The microorganisms used in this study were Gram-positive bacteria, Gram-negative bacteria, and endospores. The nanoparticles showed antimicrobial activity against Gram-positive and Gram-negative bacteria. The endospores were the microorganisms least sensitive to the metal oxide nanoparticles. The authors also attribute the bactericidal action of the nanoparticles used to the electrostatic attraction of the negatively charged pathogens and the positively charged nanoparticles [23].

According to recent literature, bacteria are able to regulate the electrical charge of their surface, which allows them to repel nanoparticles with different types of charge on their surface. The nanoparticles may have a positive, negative or neutral charge on their surface. The interaction of these three types of charge with the electric charge present on the surfaces of the bacteria in some cases seems to be the most important factor that determines the antimicrobial activity of the nanoparticles [24]. For example, Abbaszadegan et al. tested the antimicrobial activity of silver nanoparticles with a positive, negative, and neutral charge on Gram-positive (*Streptococcus pyogenes, Streptococcus mutants, and Staphylococcus aureus*) and Gram-negative (*Proteus vulgaris* and *Escherichia coli*) species. The silver nanoparticles with positive charge were the most effective against all Gram-positive and Gram-negative species. The nanoparticles with neutral charge had intermediate antimicrobial activity, and the nanoparticles with negative charge were the least effective. The authors attribute the high bactericidal activity of the Ag NPs with positive charge to the attraction of these with the negative charges present in the bacteria. Likewise, they mention that the nanoparticles with negative charge were the least effective due to the repulsion created by the equal charges between these and the bacteria. In the same study, the authors also report that *Proteus vulgaris* was the most resistant bacterium to the three types of nanoparticles used. This result suggests that some bacterial species have characteristics or mechanisms that allow them to generate resistance to the charge present on the surface of the nanoparticles [25]. This type of mechanism has been previously reported. An example of this is the development of resistance to cationic antimicrobial peptides (CAMP) in which bacteria are able to modulate the electrical charge of their surface by modifying the structure of their phospholipids [26].

These mechanisms are regulated by the envelope stress response (ESR) mechanisms. The ESR is present in Gram-positive and Gram-negative bacteria and continuously monitors biogenesis and protects the integrity of the bacterial cell envelope. The ESR can protect bacteria from contact with positively charged nanoparticles through the incorporation of D-alanine into the cell wall of Gram-positive bacteria, and reducing their overall negative net charge [27]. In Gram-negative bacteria, the ESR is involved in the modifications of the lipopolysaccharide (LPS) and its main component, the lipid-A. For example, the addition of phosphoethanolamine (PEA) to Lipid A can increase the positive charge of the LPS [28].

## 4. Defense Mechanisms against Metal Ions Release from Metal and Metal Oxide Nanoparticles

One of the antibacterial mechanisms of metal and metal oxide nanoparticles is the release of toxic ions for bacteria. For example, Wang et al. tested the antimicrobial effect of different types of metal oxide nanoparticles (Fe_2_O_3_ NPs, Fe_3_O_4_ NPs, NiO NPs, and ZnO NPs) combined with chemicals (like surfactants) on *Photobacterium phosphoreum.* They reported different effects on bactericidal activity, like additive, antagonistic and synergistic effects; and all these effects were related to the degree of ion release [29].

The industrial production of nanoparticles has increased considerably in recent years [30]. The nanoparticles produced at the industrial level are incorporated into a great variety of products, like home and garden products, appliances, automotive products, electronics, computers, food, and goods for children [31]. The accumulation of nanoparticles in the environment represents various adverse effects for the environment itself and global health [32]. Once present in the environment, the nanoparticles can be transformed [33] which would cause a change in their physicochemical properties and antimicrobial activity [34]. The different transformations that metal and metal oxide nanoparticles can undergo in the environment and that can alter their dissolution rate causing an alteration in the ion release rate are: Aggregation, ligation, redox reactions, adsorption of biomacromolecules and biologically mediated transformations [35]. In the beginning, the alteration in the dissolution rate of the metal and metal oxide nanoparticles can increase the bactericidal power of the nanoparticles due to the increased ion release rate. For example, the oxidation of Ag NPs by chloride (Cl^−^) in seawaters or growth medium increases the rate of ion release and increases its bactericidal power against *E. coli* [36]. Although initially, there is an increase in their bactericidal activity, over time, the life of the nanoparticles can be shortened with the loss of their bactericidal activity [37]. For example, oxidation and interaction with natural organic matter (NOM) of zero-valent iron nanoparticles under aerobic conditions cause the inactivation of their bactericidal properties [38].

The transformations of nanoparticles in the environment and the changes in the release rate would allow the bacteria to exert mechanisms of adaptation to the bactericidal effect of the ions released. For example, recently it was reported that efflux systems participate in the development of resistance to ions release from metal nanoparticles under non-bactericidal concentrations. Jianhua et al. exposed *Pseudomonas aeruginosa* PAO1 to different concentrations of CuO NPs, and observed an upregulation of the resistance-nodulation-cell division (RND) transporters, the P-type ATPase efflux complexes, and the cation diffusion facilitator (CDF) transporters at low (1 mg/L), medium (10 mg/L) and high (50 mg/L) CuO NPs exposure [39]. Yang et al. also exposed *Pseudomonasa aeruginosa* PAO1 to sublethal concentrations (160 nM) of intact and weathered quantum dots (QDs). The transcriptomic analysis revealed an upregulation in the transcriptional unit czcABC from the RND family efflux system [6].

These efflux complexes are expressed from metal resistance genes; these genes can be located in plasmids [40] or in the bacterial genome. In general, these genes encode for three different types of proteins families are responsible for the expulsion of ions released by the metal and metal oxide nanoparticles outside the bacterial cell. The first line of defense is formed by the members of the resistance-nodulation-cell division protein superfamily. This superfamily is formed by seven protein families that are dispersed in a great variety of microorganisms (from Archaea to Eukaryotes). RND proteins generally form efflux complexes along with two other types of proteins. The first of these two proteins is in most cases a member of the membrane fusion protein family (MFP) [41]. And the other protein belongs to the family of outer membrane factors (OMF) [42]. The efflux protein complex form by these three proteins is named CBA efflux system or CBA transporters. The RND protein is designated with the letter A, the MFP protein is designated with letter B, and the OMF protein is designated with the letter C. Some examples of these efflux complexes are: The CzcCBA efflux system that mediates the efflux of Co^2+^, Zn^2+^, Cd^2+^ and Ni^2+^, the cusCFBA efflux system that mediated the efflux of Cu^+^ and Ag^+^ and the silCFBAGP efflux system that mediates the efflux of Ag^+^. The second line of defense is formed by the members of the cation diffusion facilitators (CDF family). This family mediates the efflux of Zn^2+^, Co^2+^, Ni^2+^, Cd^2+^, and Fe^2+^ [43]. The third line of defense is formed by the members of P-type ATPases. The efflux in these transport proteins is driven by ATP hydrolysis, and these proteins mediate the efflux of H^+^, Na^+^, K^+^, Mg^2+^, Ca^2+^, Cu^+^, Ag^+^, Zn^2+^ and Cd^2+^ [44].

In addition to the efflux complexes, there are other mechanisms responsible for the protection of bacterial cells against the ions released by nanoparticles. These mechanisms include intra- and extra-cellular sequestration, bio-precipitation, enzymatic detoxification or biotransformation, pigment production and morphology alteration [45]. These mechanisms of resistance to metal and metal oxide nanoparticles have been poorly studied. In intracellular sequestration, bacteria use proteins to retain the excess of toxic metal ions in their cytoplasm. This prevents the ions from causing damage to intracellular structures and allows the bacteria to activate other defense mechanisms such as efflux pumps. For example, in bacteria with metal-resistant operons, these operons include genes that code for metal ion-sequestering proteins like *SilG* gene in the *sil* operon [46]. In extracellular sequestration, the bacteria produce ECS that immobilize the nanoparticles and prevent them from having contact with the bacteria, decreasing their bactericidal activity [47]. For example, Wang et al. reported that *E. coli* produces an ECS that reduces the specific bactericidal activity of ZnO NPs and silicon oxide nanoparticles (SiO_2_ NPs). In pigment production, some bacteria seem to produce pigments that help reduce their exposure to the ions released by the nanoparticles. For example, Ellis et al. studied the ability of three bacteria (*Staphylococcus aureus, Pseudomonas aeruginosa, and Acinetobacter baumannii*) to generate resistance to silver nanoparticles. The only bacterium that generated resistance to Ag NPs was *Pseudomonas aeruginosa*. They attribute this resistance to the production of pigments due to a color change in the colonies during the experiments. *Pseudomonas aeruginosa* produces phenazine pigments, which include pyocyanin, pyochelin, and pyoverdin. Pyocyanin can reduce Ag^+^ to Ag^0^ protecting the bacteria from the damage caused by silver ions released from nanoparticles (Figure 1) [48]. Bioprecipitation consists in the transformation of toxic metal ions into agglomerates (similar to nanoparticles) that reduce their toxicity [49]. For example, Sari et al. reported that silver ion resistant bacteria are capable of bio-precipitate silver ions into silver nanoparticles [50]. Biotransformation is the process in which toxic metals are transformed into non-toxic forms by enzymes [51]. For example, Palomo-Siguero et al. found that lactic bacteria *Lactobacillus bulgaricus* is able to enzymatically transform (using selenoenzymes like thioredoxin reductase (TRx) and glutathione peroxidase (GPx)) the chitosan-modified selenium nanoparticles (CS-SeNPs) into organic seleno-compounds like SeCys2 and SeMet [52]. In morphology alteration, the bacteria are capable of altering their morphology resulting in a lower bactericidal effect of the nanoparticles. For example, Zhang et al. reported that *E. coli* was able to develop resistance to ZnO NPs by acquiring a smaller and more oval shape [7]. All the aforementioned mechanisms are controlled by metalloregulatory and metal homeostasis systems in bacteria [53].

On the other hand, the widespread use of metal and metal oxide nanoparticles seems to stimulate the co-selection and co-expression of antibiotic resistance genes. This represents an important adverse side effect of the use of nanoparticles. Wang et al. exposed pure *E.coli* cultures and aquatic microbiota to sublethal concentrations of ZnO NPs. This type of exposure facilitated the conjugative transfer of antibiotic resistance plasmids. The exposure to sublethal concentrations of ZnO NPs increased the permeability of the cell membranes and this, in turn, increased the horizontal gene transfer frequency [54]. In a previous study Qiu et al. also exposed *E. coli* to high concentrations of titanium dioxide nanoparticles (TiO_2_ NPs), and observed that this caused a decrease in bacterial growth rate and an increase the conjugative transfer rate of antibiotic resistance genes [55].

The ESR also participates in the regulation of the mechanisms of defense against the electrical charge, ion release and the size presented by the different types of metal and metal oxide nanoparticles. The ESR in Gram-negative bacteria consists of an alternative sigma factor (RpoE or σ^E^), three different 2-component regulatory systems (like the pilus expression (Cpx) response, the regulation of capsular synthesis (Rcs) phosphorelay and the bacterial adaptive response (Bae)) and the phage shock protein response (PSP).

RpoE regulates the expression of genes involved in protein folding and degradation, cell envelope biogenesis and cell envelope modification. For example: RpoE controls the alginate production in *Pseudomonas aeruginosa*. RpoE is also involved in the modification of LPS through the PhoPQ regulon and MicL sRNAs. These modifications can increase the bacterial resistance to antimicrobial molecules like CAMP and metal or metal oxide nanoparticles. RpoE also induces the expression of RybB and MicA sRNAs. These two sRNAs downregulate the production of porins and reduces the entry pathways of the nanoparticles to the bacteria.

The first of the three different 2-component regulatory systems is Cpx. Cpx regulates the protein export systems associated with virulence. While the Rcs response (the second of the three different 2-component regulatory systems) is induced by conditions that can disrupt the cell envelope and control the production of macromolecular envelope structures and the envelope homeostasis. Finally, the bacterial adaptive response regulates the expression of different types of efflux pumps while the phage shock protein response maintains the proton motive force and prevents mislocalized secretin toxicity.

The ESR in Gram-positive bacteria is formed by three systems. The first system regulates the response to specific noxious stimuli. The second system is activated by harmful agents present in the cell wall. These systems include extracytoplasmic function (ECF) σ factors and 2-component regulatory systems. The third system is not induced by cell wall damage but does play a role in controlling cell envelope integrity. These three systems regulate the cell envelope charge, cell wall metabolism, and expression of efflux pumps [56].

## 5. Defense Mechanisms against the Production of ROS and Oxidative Stress by Metal and Metal Oxide Nanoparticles.

The generation of reactive oxygen species (ROS) during the interaction of metal and metal oxide with bacteria is another mechanism of the bactericidal activity of these types of nanoparticles. The degree of ROS generation depends on the metal or metal oxide from which the nanoparticle is synthesized, and their ion release rates. The nanoparticles like ZnO NP, CuO NP, and Ag NP present a wide range of ion release and ROS production when interacting with bacteria [57,58]. While other types of metal and metal oxide, like gold nanoparticles (Au NPs), do not appear to induce the production of ROS and its antimicrobial activity in bacteria [59]. The release of metal ions by the nanoparticles affects the respiratory chain and scavenging mechanisms. This results in production and accumulation of singlet oxygen, hydroxyl radical, hydrogen peroxide, superoxide anions, and other ROS. ROS can cause damage to the internal components of the bacteria such as structural proteins, organelles, enzymes, DNA, respiratory chain and scavenging mechanisms [60].

The ability of nanoparticles to induce ROS can be affected by their interactions with the environment in which they are used [61]. These interactions can be modulated by various factors such as the presence of light and oxygen. For example, Carré et al. reported that the photocatalytic properties of TiO_2_ NPs cause the disruption of the cell membrane of bacteria [62]. Xiu et al. reported that silver nanoparticles release ions under aerobic conditions and that this release rate increases over time. On the other hand, the Ag NPs did not show ion release under anaerobic conditions. In addition, when *E. coli* was exposed to Ag NPs under anaerobic conditions, the nanoparticles had no bactericidal effect [63]. Chen et al. exposed *P. aeruginosa* to Ag NPs under aerobic and anaerobic conditions. They observed a similar release of silver ions in the two evaluated conditions; this contradicts the reported by Xiu et al. However, the Ag NPs studied by Chen et al. also showed a lower bactericidal effect in anaerobic conditions. This is due to the fact that under anaerobic conditions there is a lower production of ROS and although the nanoparticles affect the bacterium the lack of the deleterious mechanisms induced by ROS diminishes its bactericidal activity [64]. This is important since the use of nanoparticles to fight bacteria causing diseases in anaerobic environments could cause the adaptation of bacteria and changes in microbiomes that could aggravate the pathologies [65].

Sublethal concentrations of ROS stimulates the expression of defense mechanisms in bacteria, a process which is called hormesis [66]. Hormesis causes the expression of adaptive and defense mechanisms at two different levels. The first level is the enzymatic level or short-term response. This type of response is activated with sudden changes in the intra and extracellular concentrations of ROS. It consists of the activation of the expression of ROS scavengers enzymes and allows the bacteria to maintain a balance for a few seconds or minutes. This is achieved through three different pathways. First, the enzymes responsible for the inactivating of ROS become more sensitive; in this way, as long as ROS is produced, the activity of the enzymes will be greater to inactivate them. Secondly, ROS stimulates the expression of ROS scavengers enzymes [67]. And third, adaptation can also be achieved by the increased regeneration of the bacterial ROS scavengers enzymes [68].

The second level is the long-term adaptation. The long-term adaptation is made up of two sub-levels: Transcriptional and genomic level. In the transcriptional level, ROS induces adaptation by upregulating the antioxidant mechanisms within hours to days [69]. At the genomic level, ROS can cause damage to the DNA structure, which activates DNA damage repair mechanisms. These mechanisms can be homologous recombination, excision repair, and translesion DNA synthesis. In these mechanisms, two of the DNA polymerases responsible for the translesion DNA synthesis have poor proofreading activities, so they can include incorrect bases in the DNA strands, which leads to a high frequency of spontaneous mutations and genome plasticity under adverse effects [70]. This genome plasticity can result in the development of resistance to metal and metal oxide nanoparticles [8].

**Figure 2:**
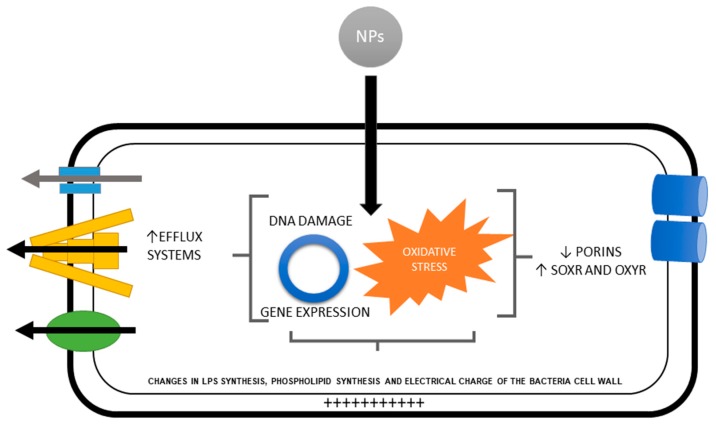
Bacterial resistance to nanoparticles is due to the change of expression of genes and the presence of oxidative stress which causes a decrease in porins, activation of SOXR and OXYR (redox-sensitive transcriptional activators), change of the electric charge of the bacterial wall, overexpression of efflux systems and change in the synthesis of lipopolysaccharides. “↑ “upregulation, “↓” downregulation.

The genes that activate the mechanisms of resistance to oxidative stress are upregulated by two regulons: SoxR and oxyR. OxyR responds to the stress induced by hydrogen peroxide, while soxR responds to superoxide anion [37]. For example, bacteria exposed to Ag NPs upregulated genes in charge of protecting against oxidative stress such as soxR, oxyR, sodA, sodB, and sodC (that produce superoxide dismutase, which degrades superoxide to hydrogen peroxide) and the katE and katG genes involved in the subsequent transformation of hydrogen peroxide into oxygen [71] (Figure 2).

## 6. Defense Mechanisms of Biofilms against Metal and Metal Oxide Nanoparticles.

In general, biofilms are a set of diverse bacteria within a matrix of extracellular polymers (ECP). The bacterial diversity presented in them results in the union of the various resistance mechanisms present in the different bacterial species to assemble a protective response against nanoparticles. Biofilms have already proven to be resistant to prolonged exposures to nanoparticles [72]. The very presence of the ECP serves as a barrier to the passage of the nanoparticles, avoiding their direct contact with the bacteria inside the biofilm [73]. The ECP of the biofilms is formed by extracellular DNA, lipids, proteins, and polysaccharides. These components interact with the nanoparticles by modifying their properties like surface charge, particle size, shape, and concentration. These modifications affect the antibacterial activity of the nanoparticles in contact with the biofilms [74], for example, electrostatic attraction between the negatively charged carboxyl groups in biofilms, and the positively charged nanoparticles like Ag NPs, ZnO NPs and SiO_2_ NPs [47,75]. Therefore, biofilms support higher concentrations of nanoparticles compared to planktonic cells. For example, Choi et al. reported the minimum bactericidal concentration of Ag NPs of 21 and 15 nm in planktonic bacteria and biofilms. The minimum bactericidal concentration was higher in the biofilm (38 mg/L) than in the planktonic bacteria (10 mg/L) [76]. The ECP of the biofilm not only modifies the nanoparticles but also has the property of binding them, preventing them from penetrating and making contact with the bacteria [47]. Peulen et al. reported that biofilms have a pore size (10–50 nm) that allows them to retain nanoparticles with sizes greater than 10 nm [76]. While, Jing et al. demonstrated that cerium oxide nanoparticles (CeO_2_ NPs) were trapped in the surface of *Pseudomonas fluorescens* and *Mycobacterium smegmatis* biofilms, while bacterial cells in the deepest part of the biofilms were not affected [77]. The degree of maturity of the biofilm also seems to influence the penetration capacity of the nanoparticles. The more mature the biofilm, the pores will be smaller and will prevent the diffusion of nanoparticles [78]. The diversity of bacterial species in biofilm result in a more heterogeneous ECP. This heterogeneity provides a more complex chemical composition to ECP and makes it more reactive in contact with nanoparticles [79].

Exposure of biofilms to non-lethal concentrations of nanoparticles can also generate hormesis processes. For example, when *Pseudomonas aeruginosa PAO1* was exposed to a nonlethal concentration of polyvinylpyrrolidone-coated silver nanoparticles (PVP-AgNPs), the bacteria presented an increase in biofilm formation, lipopolysaccharide biosynthesis, increased extracellular polymeric substances and upregulation of antibiotic resistance genes (ARGs) [80]. In another study, the sublethal concentrations of 0.5 and 2 mg/L cerium oxide nanoparticles (CeO_2_ NP) caused an increase in the production of ECP and quorum sensing through oxidative stress [81].

The bacterial diversity of the biofilms and the different mechanisms of resistance of each of the species to the nanoparticles can contribute to the resistance of the biofilm in general. For example, *Pseudomonas aeruginosa CCM 3955* and *Escherichia coli 013* have resistance to Ag NPs through the production of flagellin [16]. *Pseudomonas aeruginosa* produces pyocyanin, pyochelin, and pyoverdin to protect itself against nanoparticles [48]. While, *Enterobacter cloacae, Enterococcus sp., Klebsiella oxytoca, Klebsiella pneumoniae, Proteus mirabilis* and *Staphylococcus aureus* can express efflux complexes related to nanoparticle resistance [82].

Biofilms also have functional redundancy; this is the ability to maintain their ecosystem despite the change in their microbial composition by harmful agents, as when a biofilm loses *Competibacter spp.* (involved in phosphorus removal) due to the exposure to ZnO NPs. *Accumulibacter spp*. replaced the bacteria previously lost and its function within the biofilm [83]. The exposure of the bacterial diversity of the biofilms to non-lethal concentrations of nanoparticles also stimulates the horizontal transfer of genes and a greater diversity of resistance genes to be transferred [84].

It is estimate that biofilms cause 80% of infectious diseases, therefore, preventing the development of biofilm in clinical and industrial environments is a matter of great importance. One of the strategies in the combat of biofilms is to prevent their early stages of formation such as the adhesion of microorganisms to surfaces [85]. Various types of metal and metal oxide nanoparticles have been studied in order to prevent the adhesion of microorganisms to surfaces and avoid the formation of biofilms. For example, catheters coated with Ag NPs can prevented the formation of biofilms of *Enterococcus spp*., *Escherichia coli*, *coagulase-negative Staphylococci spp*., *Staphylococcus aureus*, *Pseudomonas aeruginosa* and *Candida albicans* on their surface [86]. Gomez-Carretero et al. integrated Ag NPs in an electrically conducting polymer layer as surface coating. This special coating was able to inhibit almost completely the formation of biofilm by Staphylococcus aureus [87]. Applerot et al. developed glass slides coated with zinc oxide nanoparticles. These glass slides had the ability to inhibit the growth of *E. coli* and *S. aureus* biofilms. In addition, the glass surfaces covered with nanoparticles had a great potential for use in industrial and medical settings [88]. Although surfaces coated with nanoparticles show promising results in the inhibition of biofilm formation, there are no studies on the possible adaptation of bacteria to this type of surfaces in the long term. Neither has been studied about the possible changes in their properties that the surfaces coated with nanoparticles can suffer in long-term clinical or industrials environments.

## 7. Future Directions

Despite the increasing knowledge on the antimicrobial activity of metal and metal oxide nanoparticles, much remains unknown about the impact of its use on the environment and global health. Scarce literature has been commissioned to study the mechanisms of adaptation of bacteria to the exposure of nanoparticles. It is important to understand these mechanisms to avoid the indiscriminate use of nanoparticles in different materials, as well as the appearance or increase of infections resistant to treatment. The authors of this paper called into action, the need for a better understanding of the mechanisms of adaptation and resistance to nanoparticles and how these can affect the environment and the health of people.

## 8. Conclusions

The authors recommend that future studies should offer more information on the mechanisms and adverse effects of the appearance of bacterial resistance to nanoparticles.

## Abbreviations

ZnO NPs	Zinc oxide nanoparticles
CuO NPs	Copper oxide nanoparticles
Fe_2_O_3_ NPs	Iron oxide nanoparticles
ECS	Extracellular substances
Ag NPs	Silver nanoparticles
MgO NPs	Magnesium oxide nanoparticles
Al_2_O_3_ NPs	Aluminum oxide nanoparticles
TiO_2_ NPs	Titanium dioxide nanoparticles
CeO_2_ NPs	Cerium oxide nanoparticles
CAMP	Cationic antimicrobial peptides
ESR	Envelope stress response
LPS	Lipopolysaccharide
PEA	Phosphoethanolamine
Cl-	Chloride
NOM	Natural organic matter
RND	Resistance-nodulation-cell division transporters
CDF	Cation diffusion facilitator transporters
QDs	Quantum dots
MFP	Membrane fusion protein family
OMF	Outer membrane factors
SiO_2_ NPs	Silicon oxide nanoparticles
Fe_3_O_4_ NPs	Ferric oxide nanoparticles
NiO NPs	Nickel oxide nanoparticles
TRx	Thioredoxin reductase
GPx	Glutathione peroxidase
CS-SeNPs	Chitosan-modified selenium nanoparticles
Cpx	Pilus expression response
Rcs	Regulation of capsular synthesis phosphorelay
Bae	Bacterial adaptive response
PSP	Phage shock protein response
ECF	Extracytoplasmic function
ROS	Reactive oxygen species
Au NPs	Gold nanoparticles
ECP	Extracellular polymers
PVP-AgNPs	Polyvinylpyrrolidone-coated silver nanoparticles
ARGs	Antibiotic resistance genes
